# Correction

**DOI:** 10.1111/cas.14598

**Published:** 2020-08-11

In an article^1^ titled “Phase 1b/2 study of blinatumomab in Japanese adults with relapsed/refractory acute lymphoblastic leukemia” by Hitoshi Kiyoi, Joan D. Morris, Iekuni Oh, Yoshinobu Maeda, Hironobu Minami, Toshihiro Miyamoto, Toru Sakura, Hiroatsu Iida, Catherine A. Tuglus, Yuqi Chen, Cedric Dos Santos, James Kalabus, Abraham Anderson, Tomoko Hata, Yasuhiro Nakashima, Yukio Kobayashi, the following changes are to be made

1. Funding Information has been changed to: Amgen and Amgen Astellas BioPharma KK.
2. Greek symbols α and γ were included after “TNF” and “IFN” in text, respectively.
3. In Section 2.3, “≤0 mg” was changed to “≤10 mg”.
4. The spelling error of "single" was corrected in Section 4, in the sentence, "Adverse events were consistent with those reported with single‐agent blinatumomab in previous adult blinatumomab studies, with grade ≥3 cytopenias occurring in a substantial proportion of patients across studies."
5. The color code was changed in Figure 3, and units were changed from mm^2^ to mm^3^. The updated figure and legend are below:

**Figure 3:**
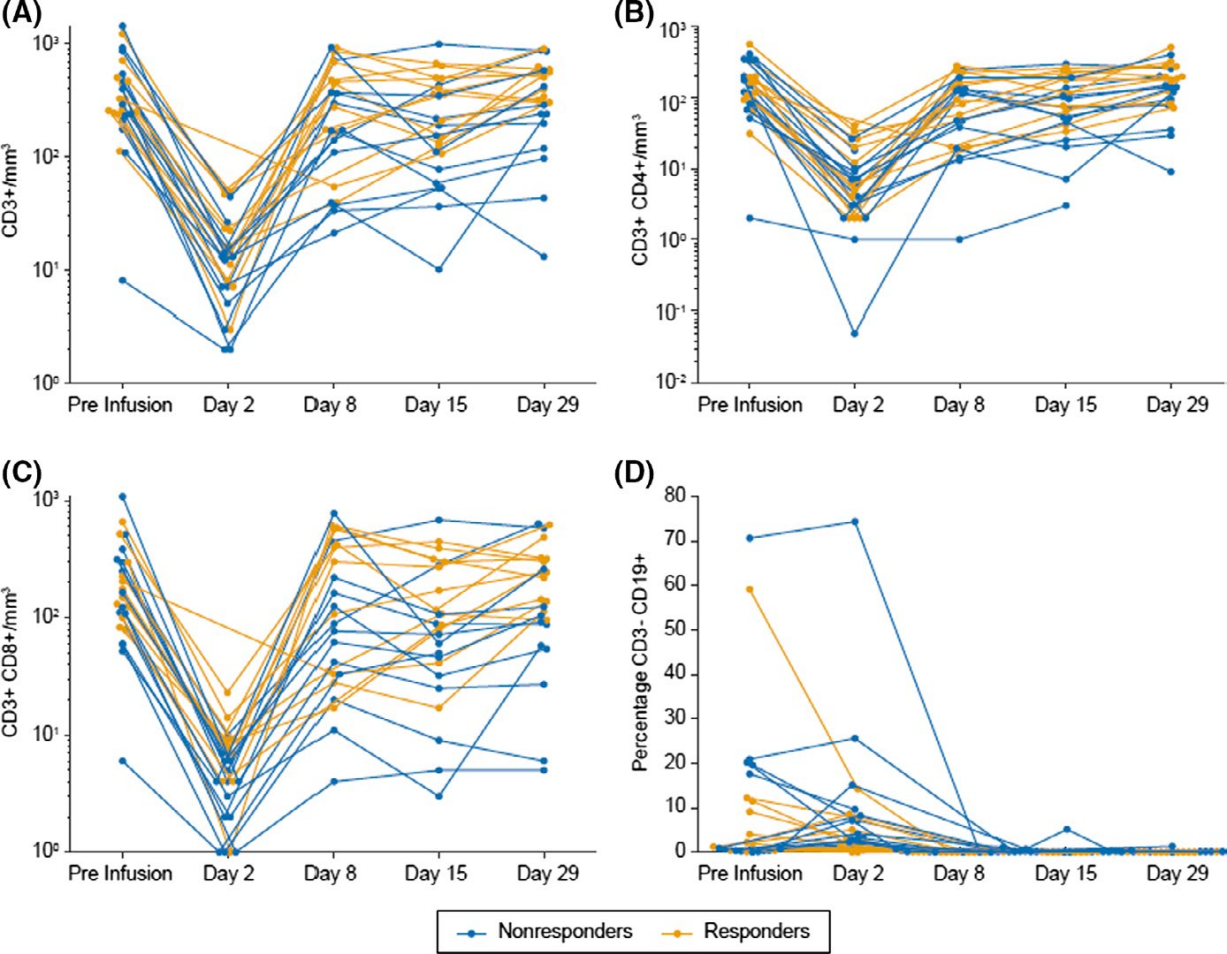
Individual patient levels of T cells and B cells during cycle 1. A, CD3+ cells/mm^3^. B, CD3+CD4+ cells/mm^3^. C, CD3+CD8+ cells/mm^3^. D, Percentage of CD3– CD19+ cells.

The authors and publisher apologize for these errors.
